# High-throughput immunophenotypic characterization of bone marrow- and cord blood-derived mesenchymal stromal cells reveals common and differentially expressed markers: identification of angiotensin-converting enzyme (CD143) as a marker differentially expressed between adult and perinatal tissue sources

**DOI:** 10.1186/s13287-017-0755-3

**Published:** 2018-01-16

**Authors:** Eliana Amati, Omar Perbellini, Gianluca Rotta, Martina Bernardi, Katia Chieregato, Sabrina Sella, Francesco Rodeghiero, Marco Ruggeri, Giuseppe Astori

**Affiliations:** 1 Advanced Cellular Therapy Laboratory, Hematology Unit, S. Bortolo Hospital, ULSS 8 Berica, Contra’ San Francesco 41, 36100 Vicenza, Italy; 2 BD Biosciences Italia, Milano, Italy; 3 Hematology Project Foundation, Vicenza, Italy

**Keywords:** Angiotensin-converting enzyme, Hematopoietic progenitor cell marker, CD143, Mesenchymal stromal cells, Flow cytometry, Bone marrow, Cord blood, Lyoplate, High-throughput screening, Immunophenotype

## Abstract

**Background:**

Mesenchymal stromal cells (MSC) are a heterogeneous population of multipotent progenitors used in the clinic because of their immunomodulatory properties and their ability to differentiate into multiple mesodermal lineages. Although bone marrow (BM) remains the most common MSC source, cord blood (CB) can be collected noninvasively and without major ethical concerns. Comparative studies comprehensively characterizing the MSC phenotype across several tissue sources are still lacking. This study provides a 246-antigen immunophenotypic analysis of BM- and CB-derived MSC aimed at identifying common and strongly expressed MSC markers as well as the existence of discriminating markers between the two sources.

**Methods:**

BM-MSC (*n* = 4) were expanded and analyzed as bulk (*n* = 6) or single clones isolated from the bulk culture (*n* = 3). CB-MSC (*n* = 6) were isolated and expanded as single clones in 5/6 samples. The BM-MSC and CB-MSC phenotype was investigated by flow cytometry using a panel of 246 monoclonal antibodies. To define the markers common to both sources, those showing the smallest variation between samples (coefficient of variation of log_2_ fold increase ≤ 0.5, *n* = 59) were selected for unsupervised hierarchical cluster analysis (HCL). Differentially expressed markers were identified by directly comparing the expression of all 246 antigens between BM-MSC and CB-MSC.

**Results:**

Based on HCL, 18 markers clustered as strongly expressed in BM-MSC and CB-MSC, including alpha-smooth muscle antigen (SMA), beta-2-microglobulin, CD105, CD13, CD140b, CD147, CD151, CD276, CD29, CD44, CD47, CD59, CD73, CD81, CD90, CD98, HLA-ABC, and vimentin. All except CD140b and alpha-SMA were suitable for the specific identification of ex-vivo expanded MSC. Notably, only angiotensin-converting enzyme (CD143) was exclusively expressed on BM-MSC. CD143 expression was tested on 10 additional BM-MSC and CB-MSC and on 10 umbilical cord- and adipose tissue-derived MSC samples, confirming that its expression is restricted to adult sources.

**Conclusions:**

This is the first study that has comprehensively compared the phenotype of BM-MSC and CB-MSC. We have identified markers that could complement the minimal panel proposed for the in-vitro MSC definition, being shared and strongly expressed by BM- and CB-derived MSC. We have also identified CD143 as a marker exclusively expressed on MSC derived from adult tissue sources. Further studies will elucidate the biological role of CD143 and its potential association with tissue-specific MSC features.

**Electronic supplementary material:**

The online version of this article (doi:10.1186/s13287-017-0755-3) contains supplementary material, which is available to authorized users.

## Background

Mesenchymal stromal cells (MSC) are a heterogeneous population of nonhematopoietic multipotent progenitor cells capable of self-renewal and differentiation into mesodermal cell lineages [1]. MSC can be isolated from various human tissues [2–4], where they may be recognized as pericytes and function as a source of cells for tissue repair and regeneration [5, 6]. Although bone marrow (BM) remains the most widely recognized source of MSC for clinical use, the invasive procedure of BM collection has increased interest for other MSC sources. In this context, cord blood (CB) has emerged as an alternative to BM, being collected noninvasively and without major ethical concerns since it is commonly discarded as a medical waste [7, 8]. Given the low frequency of MSC progenitors within CB, CB-derived MSC have been mostly isolated as single clones, showing a small spindle-shaped morphology and unique differentiative and proliferative properties, together with a normal karyotype after prolonged expansion [7].

Currently, the widely adopted MSC definition relies on three minimal criteria according to the International Society for Cellular Therapy (ISCT), namely: adherence to plastic under standard culture conditions; expression of CD105, CD73, and CD90, and lack of expression of hematopoietic and endothelial surface markers CD14, CD45, CD34, CD11b, HLA-DR, and CD31; and in-vitro differentiation potential into osteocytes, chondrocytes, and adipocytes under appropriate culture conditions [9]. These criteria still represent the accepted standards for the scientific community to characterize human MSC, despite functional and phenotypic differences that may exist across tissue sources, culture conditions, and extent of ex-vivo expansion [10]. For instance, an impaired adipogenic potential of MSC derived from perinatal tissues as CB-MSC is well documented, likely due to their intermediate state between adult and embryonic stem cells [11, 12]. Similarly, the stromal vascular fraction of adipose tissue (AT) meets the negativity requirements for CD45 and CD31, but not for CD34 which is expressed at variable levels during the early stages of culture [13, 14]. Beyond these observations, MSC characterization remains largely confined to the above-described criteria, further complicated by the lack of unique and definitive cell surface markers.

At present, flow cytometry represents the gold standard clinical tool for studying the immunophenotype of ex-vivo expanded MSC as part of quality assessment for the release of MSC produced in compliance with good manufacturing practice (GMP) standards. Recent advances in high-throughput flow cytometry allow us to profile hundreds of human cell surface markers in a single assay, thus facilitating an efficient and comprehensive analysis of the MSC surface proteome. However, very few studies have investigated the in-depth MSC immunophenotype using this approach [15–17] and, to the best of our knowledge, comparative studies comprehensively characterizing the MSC phenotype across several tissue sources are still lacking.

The present study provides a comparative and comprehensive 246-antigen immunophenotypic analysis, performed with the aim of defining common and differentially expressed markers between BM- and CB-derived MSC. The high-throughput screening approach, combined with a rigorous method of marker selection, allowed us to uncover new common markers for defining MSC, regardless of the source and of other variables potentially influencing MSC phenotype. On the other hand, the exclusive recognition of CD143 as a marker able to segregate adult from perinatal MSC may facilitate future research towards the identification of functional differences that may impact MSC efficacy in vivo.

## Methods

### Isolation and ex-vivo expansion of MSC from adult and perinatal sources

MSC were isolated and expanded from BM, CB, AT, and umbilical cord (UC). For BM, UC, and AT, collection procedures were approved by the ethics committee of S. Bortolo Hospital, Vicenza, Italy (Act 40/09 of 16 December 2009). For CB, informed consent was received from the mothers (IBMDR SCO101, version 2, January 2013 and reference protocol SIT-VR 13/73).

BM-MSC were generated from washouts of discarded human BM collection bags and filters (*n* = 4, median donor age 31.5 years, range 20–47 years, two males and two females) after two washing steps with 200 ml saline and centrifugation at 2000 rpm for 10 min.

Briefly, whole unprocessed total nucleated cells (TNC) were plated at the concentration of 10 × 10^4^ cells/cm^2^ in low-glucose Dulbecco’s modified Eagle’s medium (DMEM) supplemented with 10% heat-inactivated fetal bovine serum (FBS) (both from Gibco, Thermo Fisher Scientific, Waltham, USA), 100 U/ml penicillin, and 100 μg/ml streptomycin (Sigma Aldrich, Saint Louis, Missouri, USA). Cultures were kept at 37 °C in a humidified 5% CO_2_ atmosphere. After 72 h, nonadherent cells were removed, and fresh medium was added (passage (P)0). The resulting plastic-adherent cells were fed twice a week, harvested at 80–90% confluence using 10× TrypLE™ Select (Gibco, Thermo Fisher Scientific), and then subcultured at a lower density (2000 cells/cm^2^) for 2–3 additional passages.

**Fig. 1:**
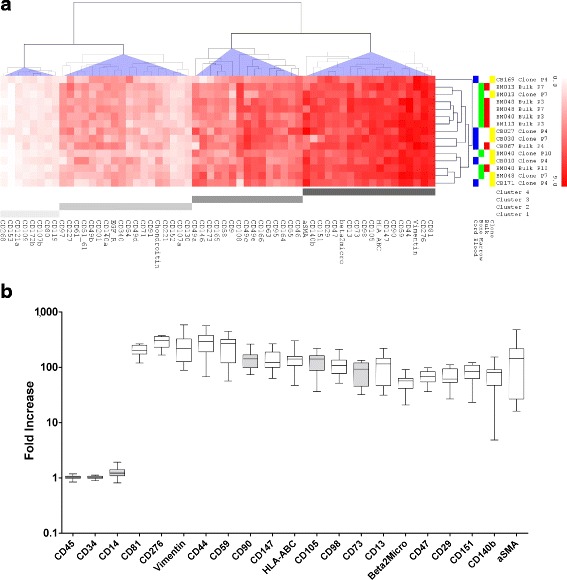
Common MSC marker identification. **a** Marker classification according to unsupervised HCL. Heat-map expression of the 59 markers selected by HCL on both bone marrow (BM)- and cord blood (CB)-MSC samples. Antigen expression level is color coded from white (no expression) to red (strong expression). Data are presented as log_2_ FI (median fluorescence intensity on the isotype control). Markers are shown according to four clusters of expression (no expression: cluster 1; low expression: cluster 2; intermediate expression: cluster 3; strong expression: cluster 4), with clusters identified with different shades of gray; CB-MSC samples are shown in blue; BM-MSC samples in green; bulk samples in red; single clones in yellow. **b** Common MSC marker identification. Boxes extend from 25th percentile to the 75th percentile, the line in the middle represents the median value and the whiskers extend from minimum to maximum values. ISCT-positive and negative markers are shown in grey. Abbreviations: HCL, hierarchical clustering; FI, fold increase

For the study, nine BM-MSC cultures were generated from four donors. Cells were expanded and analyzed as “bulks”—i.e., as a mixture of more colonies isolated as a whole from the same donor (*n* = 6, two of which were analyzed at two different passages)—or as single clones isolated from the bulk culture (*n* = 3, Figs. 1 and 3). In these selected experiments, individual MSC clones were isolated and picked up from the bulk culture using 10-mm × 10-mm cloning cylinders (Merck Millipore, Darmstadt, Germany). MSC clones were isolated from the surrounding cells and then dissociated within the cylinder, resuspended, transferred to a new vessel as a pure colony, and subcultured.

CB-MSC cultures were established from six donors (*n* = 6, four males and two females) as described previously by our group [7]. Briefly, mononuclear cells (MNC) were obtained by density gradient centrifugation (Lymphoprep™, Sentinel Ch. Spa, Milan, Italy) of whole CB diluted 1:1 with phosphate-buffered saline (PBS; Sigma-Aldrich), and cultured in low-glucose DMEM supplemented with 20% FBS, 10^–7^ M dexamethasone (Hospira, Illinois, USA), 100 U/ml penicillin, and 100 μg/ml streptomycin, at a density of 1–2 × 10^6^ cells/cm^2^ and 5–7 × 10^6^ cells/ml. Cells were incubated at 37 °C in a humidified atmosphere containing 5% CO_2_. After 1 week from initial plating, nonadherent cells were removed. The remaining cells were fed once a week in the absence of dexamethasone and checked for colony appearance for a maximum of 4 weeks. When reaching 80% confluence, cells were harvested using 10× TrypLE™ Select and subcultured at a density of 4000 cells/cm^2^. The medium was replaced twice a week and proliferation patterns were established by counting cells each week. For the study, CB-MSC from six independent donors were yielded and expanded from the starting material as single clones in five cases and as a combination of a few clones in one case.

AT- and UC-derived MSC were isolated as described previously by our group [18, 19]. Fresh UC was collected from the Obstetrics and Gynecology Unit of S. Bortolo Hospital (Vicenza, Italy) from full-term deliveries after cesarean section. About 20–30 cm of the whole UC was rinsed with PBS in presence of 100 U/ml penicillin and 100 μg/ml streptomycin, minced in a Petri dish into small fragments of about 2–3 mm, transferred to a 75-ml cell culture flask, and digested with 0.1% collagenase type II (Gibco, Thermo Fisher Scientific) for 4 h at 37 °C in a humidified atmosphere containing 5% CO_2_. An equal volume of 0.25% trypsin–EDTA (Sigma-Aldrich) was added for 30 min. The enzyme’s action was blocked with low-glucose DMEM supplemented with 10% FBS, 100 U/ml penicillin, and 100 μg/ml streptomycin, and cultures were kept at 37 °C in a humidified atmosphere containing 5% CO_2_. After 4 days, tissue fragments were removed and the flasks were rinsed twice with PBS, and then complete fresh medium was added. The resulting plastic-adherent cells were fed twice a week and harvested at 80–90% confluence using 10× TrypLE™ Select, and then subcultured at a density of 1500–2000 cells/cm^2^.

AT-MSC were obtained from the adipose tissue of healthy subjects (*n* = 10) undergoing abdominal plastic surgery. About 100–150 cm^2^ of AT was washed with PBS in the presence of 100 U/ml penicillin and 100 μg/ml streptomycin to eliminate blood contamination. For the isolation of the stromal vascular fraction, AT was cut and digested with 0.1% collagenase type II for 1 h at 37 °C in a humidified atmosphere containing 5% CO_2_. The collagenase activity was then neutralized with 10 ml low-glucose DMEM. After centrifugation at 140 × g for 10 min, the cell pellet was resuspended, filtered through a 70-μm nylon cell strainer (Falcon®, Corning, Corning, NY, USA) and centrifuged at 270 × g for 4 min. The obtained stromal vascular fraction was plated into F75 cell culture flasks in the presence of low-glucose DMEM containing 10% FBS, 100 U/ml penicillin, and 100 μg/ml streptomycin, and then cultures were kept at 37 °C in a humidified atmosphere containing 5% CO_2_. After 3 days, nonadherent cells were removed and complete fresh medium was added. The resulting plastic-adherent cells, termed AT-MSC, were cultured until 80–90% confluent, and then harvested using 10× TrypLE™ Select and subcultured at a density of 1500–2000 cells/cm^2^.

Cryopreserved MSC from all sources were used for later experiments. MSC batches with a viability of 90% or more were considered for flow cytometry analysis.

### Lyoplate staining

The BM- and CB-MSC phenotype was investigated using the BD Lyoplate™ Human Cell Surface Marker Screening Panel (BD Biosciences, Cat. 560747, San Jose, CA, USA), a system consisting of three 96-well plates, each well containing a lyophilized unconjugated antibody specific for a cell surface protein for a total of 242 monoclonal antibodies (mAbs). The panel also contains mouse and rat isotype controls for assessing the isotype-specific background. Additional purified or fluorochrome-conjugated mouse anti-human mAbs and related isotype or negative controls were added to the original panel, including purified anti-chondroitin sulfate (9.2.27 clone; BD Pharmingen™), allophycocyanin (APC)-anti-CD276 (FM276 clone; Miltenyi Biotec, Bergisch-Gladbach, Germany), Alexa Fluor® 488-anti-vimentin (RV202 clone; BD Pharmingen™), and APC-anti-α-Smooth Muscle Actin (αSMA, clone #1A4; R&D Systems, Minneapolis, MN, USA). Isotype controls included mouse IgG2A (clone #20102; R&D Systems) and mouse IgG2B (clone IS6-11E5.11; Miltenyi Biotec) APC-conjugated mAbs. BM- and CB-MSC cell staining was performed according to the manufacturer’s recommendations with slight modifications. About 40,000 cells in each well were suspended in 100 μl buffer containing PBS 1% MACS bovine serum albumin (BSA) Stock Solution (Miltenyi Biotec) and 5 mM EDTA. Except for fluorochrome-conjugated mAbs, antibody binding was detected using Alexa Fluor® 647-conjugated goat anti-mouse Ig and goat anti-rat Ig secondary antibodies. Intracellular markers (chondroitin sulfate, vimentin, and αSMA) with related controls were detected using the BD Intrasure™ Kit, according to the manufacturer’s recommendations. Samples were acquired (4000 events) using BD FACSCanto II (BD-Bioscience). Data were analyzed by FlowJo software (Treestar, Ashland, OR, USA).

### Lyoplate data analysis

Fold increase of the median fluorescence intensity with respect to the isotype control (FI), the percentage of positive cells (%pos), and robust coefficient of variation (rCV) were used to describe each marker.

Log_2_ transformation of FI was performed to improve the resolution of low FI values.

Subsequently, to define common markers to BM- and CB-MSC, those showing the smallest variation between samples (CV of log_2_ FI ≤ 0.5, *n* = 59) were selected (Additional file 1: Figure S1) for unsupervised hierarchical cluster analysis (HCL). Mean and CV values of log_2_ FI for each marker are reported in Additional file 2 (Table S1).

The log_2_ FI values of markers with low variation among samples were analyzed using MeV software (TM4 Software Development). Unsupervised HCL was performed using Euclidean distances as the distance metric and the complete linkage as linkage method of analysis [20].

### Flow cytometry analysis of angiotensin-converting enzyme (ACE) (CD143) expression

The expression of ACE was analyzed on 10 additional samples for each source (BM, CB, UC, AT) using phycoerythrin (PE) mouse anti-human angiotensin converting enzyme (CD143) (clone BB9) and PE mouse IgG1 isotype control (clone MOPC-21), both purchased from BD Pharmingen™. One hundred thousand cells were stained for 15 min at room temperature in the dark with the specific antibody or isotype control. At least 5000 events in the morphological MSC gate were acquired on a BD FACSCanto II (BD Biosciences). Data were analyzed by FlowJo software in terms of FI and %pos.

### Statistical analysis

The expression of all 246 markers was compared using Mann-Whitney *U* test. Each marker was considered differentially expressed between BM- and CB-MSC if the Mann-Whitney *p* value was ≤ 0.01. In addition, the differences in ACE (CD143) expression between BM-, UC-, CB-, and AT-MSC were computed by Mann-Whitney *U* test.

## Results

### Identification of markers commonly expressed on MSC

**Fig. 2:**
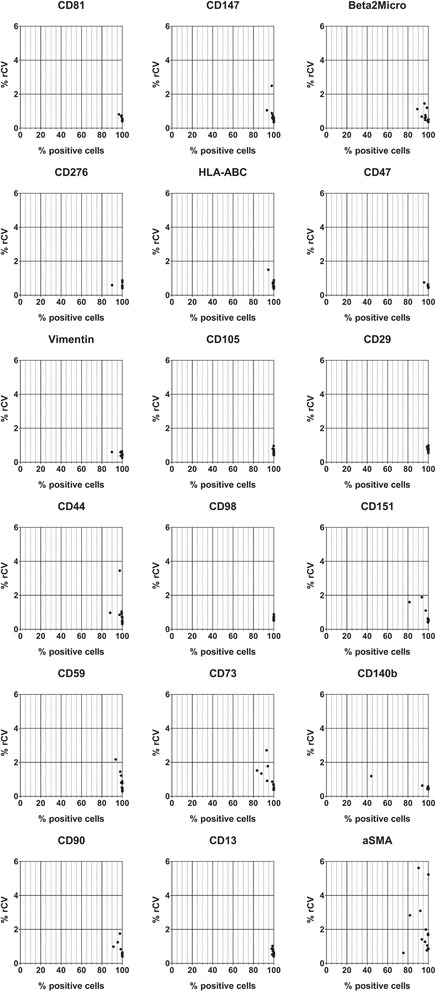
Marker refinement according to % of positive cells and robust coefficient of variation (rCV). The common and highly expressed markers were further selected according to a % of positivity higher than 80% and a rCV < 4% for each sample

Starting from 246 antigens and proceeding to an unsupervised hierarchical classification of those showing the lowest variation across samples in terms of CV (*n* = 59), we identified four groups of markers according to their relative expression intensity (Fig. 1a and Additional file 3: Table S2). A total of 18 markers clustered as strongly expressed and showed log_2_ FI values higher than 5.6, meaning that they had a median fluorescence intensity at least 48.5 times higher when compared to the isotype controls or the negative markers (Fig. 1b). Beyond CD90, CD105, and CD73, this included CD44, CD13, CD29, HLA-ABC, vimentin, beta-2-microglobulin, CD147, CD151, CD276, CD47, CD59, CD81, CD98, CD140b, and alpha-SMA. These markers were found consistently expressed by both BM- and CB-MSC (*n* = 9 and *n* = 6, respectively), regardless of sample heterogeneity ascribable to tissue source or passage history. Furthermore, none of them was found to be differentially expressed when comparing bulk cultures with the derived clones (*n* = 3), differently to other antigens (CD57, CD35, CD6, CD21, CD25, CD34, CD62E, CD62L, CD43, CD33, CD56, and CD229) which, however, did not fall in the group of common MSC markers (Additional file 4: Figure S2). Analysis was further refined on the markers having the highest percentage of positivity and the narrowest distribution for each sample. Overall, all the identified markers apart from CD140b and alpha-SMA showed a % of positivity close to 100% and a very low % rCV in most samples, thus being suitable for the identification of in-vitro expanded MSC (Fig. 2). Conversely, the expression of alpha-SMA was characterized by a high donor-to-donor heterogeneity and CD140b showed high positivity in all samples except one (Fig. 2).

### Identification of markers differentially expressed on BM-MSC and CB-MSC

**Fig. 3:**
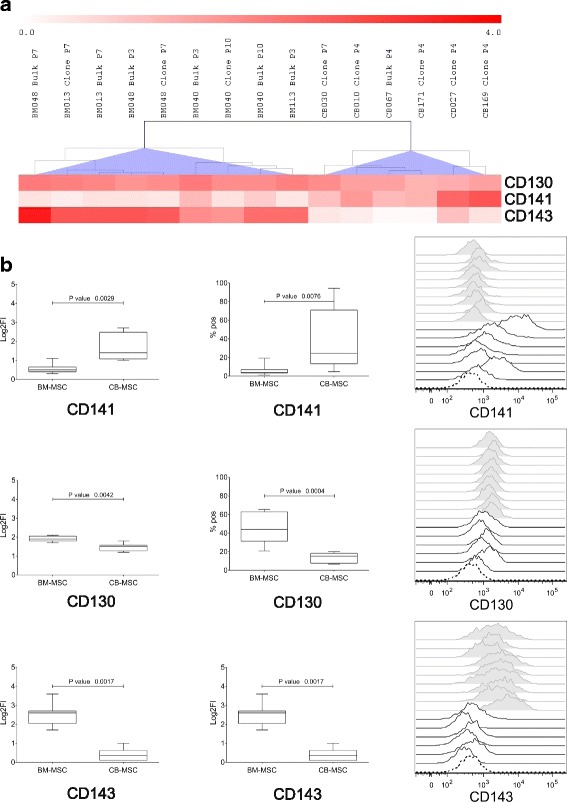
Differentially expressed markers between bone marrow mesenchymal stromal cells (BM-MSC) and cord blood mesenchymal stromal cells (CB-MSC). **a** Heat-map expression of the three differentially expressed markers: CD130, CD141, and hematopoietic progenitor cell marker (CD143/ACE). Antigen expression level is color coded from white (no expression) to red (very strong expression). **b** Boxes of expression extend from 25th percentile to the 75th percentile, the line in the middle represents the median value and the whiskers extend from minimum to maximum values. The boxes referred to the log_2_ fold increase (Log_2_FI) and % of positive (% pos) cells. The differences were computed by Mann-Whitney *U* test, *p* < 0.01. Mean fluorescence intensity histograms of each marker are reported on the right. White histograms: BM-MSC; grey histograms: CB-MSC

**Fig. 4:**
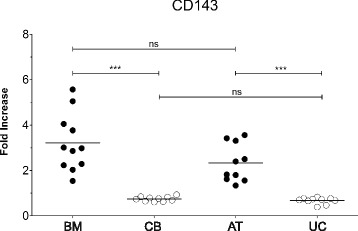
CD143 verification as a discriminating marker between adult and perinatal MSC sources. Dot plots of CD143 expression as FI in all the investigated MSC sources (adipose tissue (AT), umbilical cord (UC), bone marrow (BM), and cord blood (CB)). The differences were computed by Mann-Whitney *U* test, ***p < 0.0001. Black circles: adult MSC sources; white circles: perinatal MSC. ns not significant

In order to uncover determinants able to segregate MSC according to origin, the expression of the starting 246 antigens was compared between BM- and CB-MSC. CD130, CD141, and CD143 showed significant expression differences between the sources (*U* Mann-Whitney cut-off *p* value ≤ 0.01, Fig. 3a and b). Among them, CD141 was expressed only on CB-MSC, but at variable levels. CD130 was expressed by BM-MSC, although also at low levels by CB-MSC. Importantly, ACE (CD143) was constantly expressed solely by BM-MSC. Therefore, this latter marker only clearly distinguished between the two MSC sources. The exclusive surface localization of CD143 on BM-MSC was further verified on 10 additional MSC samples for each source, further confirming previous data (*p* < 0.0001, Fig. 4).

### Evidence of CD143 as a discriminating marker between adult and perinatal MSC

The analysis of CD143 expression on AT- and UC-MSC (*n* = 10 for each MSC source) confirmed the presence of CD143 only on the surface of AT-MSC (*p* < 0.0001, Fig. 4), endorsing the hypothesis of its exclusive expression on MSC derived from adult sources. These data therefore support a role for CD143 as a discriminating marker between adult and perinatal MSC sources.

## Discussion

Bone marrow remains the primary MSC source for clinical applications even if its collection is characterized by an invasive procedure. Therefore, the search for alternative sources has increased over the past years. In this regard, a comprehensive characterization of MSC isolated and expanded ex vivo from alternative sources is essential to unravel potential functional differences that may impact the in-vivo efficacy of MSC [21]. To that purpose, MSC heterogeneity within and between the several tissue sources needs to be properly investigated and its assessment not limited to measuring the level of expression of the classical surface markers.

In the present study we used a high-throughput flow cytometry-based screening approach to investigate the immunophenotype of both BM- and CB-derived MSC. The first endpoint was to find new common markers able to complement the existing minimal panel for MSC characterization.

Starting from 246 antigens and proceeding to an unsupervised hierarchical classification of the markers with the lowest variation across the samples, we came to select just 18 (7.3%) strongly expressed markers. These markers were shared and consistently expressed by all MSC samples regardless of the source, the frequency of MSC clones in the starting material, or the extent of ex-vivo expansion, thus being independent of all the variables that could influence MSC phenotype. In this regard, the peculiarity of our analysis resided in the use of a stringent CV cut-off of log_2_ FI as a screening method to define a common set of markers uniformly expressed by MSC cultures differently isolated and expanded.

Beyond CD90, CD105, and CD73 and other markers known to be associated with MSC phenotype, such as CD29, CD44 CD13, HLA-ABC, and vimentin, several nonclassical markers were found constantly expressed at high levels by both BM- and CB-MSC. These markers, including CD59, CD81, CD47, CD276, CD151, CD147, CD98, and beta-2-microglobulin, are involved in various nonspecific biological processes such as immune activation or inhibition and epithelial-to-mesenchymal transition [22–26]. Their similarity with the expression patterns of classical MSC markers, resulting from the analysis of % of positive cells and rCV, particularly supports their suitability as additional reference markers for the specific identification of MSC. The consistent expression of nonclassical markers on the surface of AT-MSC is also supported in the literature [15, 17] using a 242-antigen high-throughput screening analysis. It should be mentioned that the other authors detected a wealth of other molecules not distinguished by us; this discrepancy may be partially due to the restrictive marker selection applied in the present study.

Okolicsanyi and co-authors demonstrated that commercially available human MSC co-express MSC and neural markers during extended culture [27]. Such neural markers, reported to influence neural differentiation, were CD304, CD271, CD200, CD146, CD73, CD56, and CD24. Except for CD304, not included in our 246-antigen screening panel, only CD73 and CD146 clustered as strong and intermediate markers, respectively, while the others did not fall into the group of the 59 antigens selected in the present study as the less variable markers for BM- and CB-MSC. Therefore, this neural panel did not allow us to identify subgroups of MSC samples (Additional file 5: Figure S3).

The pericyte markers CD140b and alpha-SMA appeared to be not consistently expressed across all donors, despite being shared at high levels by both MSC sources. Hence, their role in MSC characterization is currently under investigation. It is conceivable that CD140b represents a relevant marker for MSC, being identified together with CD276 by Camilleri and co-authors [28]. This latter marker was found by the authors constitutively expressed at robust levels on AT-MSC.

Besides the identification of common and strongly expressed markers for MSC, an important outcome of the present study was to find the existence of markers differentially expressed among sources. Increasing evidence in this regard suggests that MSC from different origins may have differences in marker expression and inherent properties [21, 29]. Our comparative analysis unraveled only 3/246 (1.2%) markers differentially expressed between BM- and CB-MSC, specifically CD130, CD141, and CD143. CD130 was more represented on BM-MSC, although expressed at low levels also by CB-MSC, while CD141 was expressed only by CB-MSC, but not consistently across samples. Conversely, the expression of CD143 was exclusively traceable at intermediate levels on BM-MSC in all the investigated samples. The observed high variability in CD130 and CD141 expression, corroborated by literature data on cultured AT-MSC [16, 17, 29], led us to consider these markers as being not eligible for further investigation. On the other hand, the consistent surface localization of CD143 only on BM-MSC encouraged the hypothesis, further verified on an independent set of 10 samples for each source, of a role for this marker in discriminating BM-MSC from CB-MSC. Furthermore, its exclusive recognition on AT-MSC but not on UC-MSC allowed us to extend ACE/CD143 as a marker able to segregate adult from perinatal MSC.

ACE/CD143, also known as hematopoietic progenitor cell marker, is a zinc-dependent carboxydipeptidase member of the renin-angiotensin system, with broad substrate specificity and a well-recognized role in blood pressure regulation and vascular remodeling [30]. Moreover, ACE/CD143 is involved in the development and regulation of hematopoiesis [31–33]. Zambidis et al. first demonstrated that the enzymatic activity of ACE, producing angiotensin II, is required for hemangioblast expansion and differentiation into either blood or endothelial cells, through the modulation of angiotensin II-binding receptors [34]. The monoclonal antibody BB9, raised initially to human BM stromal cells [35], recognizes the somatic form of ACE as a marker of human hematopoietic stem cells in human embryonic, fetal, and adult hematopoietic tissues at all stages of hematopoietic ontogeny [36]. BB9 was found to react with a subpopulation of CD34^+^ACE^+^ cells capable of sustaining multilineage hematopoietic cell engraftment in adult BM, and mobilized peripheral blood, fetal liver, and umbilical CB after transplantation in NOD/SCID mice [35–37]. Therefore, the expression of ACE accompanies the emergence of hematopoiesis in all blood-forming tissues, starting from the human embryo where the marker identifies pre-hematopoietic mesodermal precursors ACE^+^CD34^–^CD45^–^ responsible for definitive hematopoiesis [37].

Our observation that CD143 is constitutively expressed by BM-MSC is in accord with the reactivity of BB9 with BM stromal cells [35], and therefore corroborates the hypothesis of a locally active renin-angiotensin system within the BM. The presence of CD143 on the surface of adult AT-derived MSC agrees with the observations of Matsushita et al. [38, 39] describing that endogenous angiotensin II production is increased in MSC undergoing adipocyte differentiation via increased local renin expression, suggesting that endogenous angiotensin II secreted by MSC and differentiated adipocytes contributes to the modulation of adipogenesis. This observation is further supported by the scarce propensity to generate adipose tissue exhibited by CB-MSC [11, 12].

The absence or presence of ACE/CD143 on different MSC sources is intriguing and could unravel different MSC properties. Together with a suggested role of CD143 in hematopoiesis and in MSC capacity to drive adipogenesis, Silva and co-authors reported that low expression of ACE can be used as a biomarker to identify an endothelial cell subpopulation that is more capable of driving neovascularization [40]. Further studies are required to better understand the significance of CD143 expression and its potential association with tissue-specific MSC features. Certainly, since no marker able to predict the in-vivo efficacy of MSC has been identified, the demonstration of a functional role of CD143 may be of considerable clinical impact and would allow a more careful selection of MSC-based products according to the clinical goals.

## Conclusions

To the best of our knowledge, this is the first study that comprehensively compared the phenotype of BM-MSC and CB-MSC. What emerges is a shared and strong expression of 18 markers; eight of them are nonclassically associated with the MSC phenotype but consistently expressed regardless of tissue harvest, clonal isolation, or extent of ex-vivo expansion, and therefore could be considered as additional reference markers for MSC.

Besides an in-depth characterization involving 246 different markers, this study also provides a rigorous method of analysis that allowed us to select only the markers with the smallest variation across heterogeneous samples, thus overcoming any bias potentially influencing MSC phenotype.

The main result of the work is the exclusive recognition of ACE/CD143 as a marker able to segregate adult from perinatal MSC, being CD143 unequivocally expressed on BM- and AT-MSC but not on UC- and CB-MSC. This finding may facilitate future research towards the identification of functional differences between the MSC sources that may impact their in-vivo efficacy.

## Additional files

Additional file 1: Figure S1. Transformation and selection. a) The log_2_ transformation of FI was performed to improve the resolution of low FI values. b) The markers with the smallest variability across samples independently from the source were selected according to a CV of log_2_ FI ≤ 0.5. Accordingly, 59 markers represented below the dotted line were subsequently considered for unsupervised hierarchical cluster analysis. (PDF 439 kb)

Additional file 2: Table S1. Mean and CV values of log_2_ FI for each of 246 investigated markers. (DOCX 22 kb)

Additional file 3: Table S2. List of the lowest variable markers (*n* = 59, CV cut-off of log_2_ FI ≤ 0.5) sorted by function and cluster expression. Markers with no expression were assigned to cluster 1, low-expressed markers to cluster 2, and intermediate markers to cluster 3, while a total of 18 markers clustering as very strong were assigned to cluster 4. (PPTX 50 kb)

Additional file 4: Figure S2. Differentially expressed markers between bulk cultures and single derived clones. The expression of each marker (*n* = 246) was compared by paired *t* test (*n* = 3; *p* < 0.01). (TIFF 1629 kb)

Additional file 5: Figure S3. Neural marker classification according to unsupervised HCL. Heat-map expression of the markers identified by Okolicsanyi [27]: CD24, CD200, CD271, CD56, CD146, and CD73. Antigen expression is color coded from white (no expression) to red (strong expression). Data are presented as log_2_ FI (median fluorescence intensity on the isotype control). (TIFF 1568 kb)

## Abbreviations

ACE: Angiotensin-converting enzyme
APC: Allophycocyanin
AT: Adipose tissue
BM: Bone marrow
BSA: Bovine serum albumin
CB: Cord blood
DMEM: Dulbecco’s modified Eagle’s medium
FBS: Fetal bovine serum
FI: Fold increase
HCL: Hierarchical cluster analysis
ISCT: International Society for Cellular Therapy
mAb: Monoclonal antibody
MNC: Mononuclear cells
MSC: Mesenchymal stromal cells
PBS: Phosphate-buffered saline
PE: Phycoerythrin
rCV: Robust coefficient of variation
TNC: Total nucleated cells
UC: Umbilical cord
SMA: Smooth muscle antigen

## References

[1] Pittenger MF (1999). Multilineage potential of adult human mesenchymal stem cells. Science.

[2] Erices A, Conget P, Minguell JJ (2000). Mesenchymal progenitor cells in human umbilical cord blood. Br J Haematol.

[3] Zuk PA (2002). Human adipose tissue is a source of multipotent stem cells. Mol Biol Cell.

[4] Capelli C (2011). Minimally manipulated whole human umbilical cord is a rich source of clinical-grade human mesenchymal stromal cells expanded in human platelet lysate. Cytotherapy.

[5] Da Silva ML, Caplan AI, Nardi NB (2008). In search of the in vivo identity of mesenchymal stem cells. Stem Cells.

[6] Caplan AI (2016). MSCs: the sentinel and safe-guards of injury. J Cell Physiol.

[7] Amati E (2017). Generation of mesenchymal stromal cells from cord blood: evaluation of in vitro quality parameters prior to clinical use. Stem Cell Res Ther.

[8] Zhang X (2011). Isolation and characterization of mesenchymal stem cells from human umbilical cord blood: reevaluation of critical factors for successful isolation and high ability to proliferate and differentiate to chondrocytes as compared to mesenchymal stem cells from bone marrow and adipose tissue. J Cell Biochem.

[9] Dominici M (2006). Minimal criteria for defining multipotent mesenchymal stromal cells. The international society for cellular therapy position statement. Cytotherapy.

[10] Reinisch A (2015). Epigenetic and in vivo comparison of diverse MSC sources reveals an endochondral signature for human hematopoietic niche formation. Blood.

[11] Abdulrazzak H (2010). Biological characteristics of stem cells from foetal, cord blood and extraembryonic tissues. J R Soc Interface.

[12] Ragni E (2013). Adipogenic potential in human mesenchymal stem cells strictly depends on adult or foetal tissue harvest. Int J Biochemistry Cell Biol.

[13] Bourin P (2013). Stromal cells from the adipose tissue-derived stromal vascular fraction and culture expanded adipose tissue-derived stromal/stem cells: a joint statement of the international federation for adipose therapeutics and science (IFATS) and the international society for cellular therapy (ISCT). Cytotherapy.

[14] Astori G (2007). “In vitro” and multicolor phenotypic characterization of cell subpopulations identified in fresh human adipose tissue stromal vascular fraction and in the derived mesenchymal stem cells. J Transl Med.

[15] Donnenberg AD (2015). The cell-surface proteome of cultured adipose stromal cells. Cytometry A.

[16] Baer PC (2013). Comprehensive phenotypic characterization of human adipose-derived stromal/stem cells and their subsets by a high throughput technology. Stem Cells Dev.

[17] Walmsley GG (2015). High-throughput screening of surface marker expression on undifferentiated and differentiated human adipose-derived stromal cells. Tissue Eng Part A.

[18] Chieregato K (2012). A study on mutual interaction between cytokine induced killer cells and umbilical cord-derived mesenchymal cells: implication for their in-vivo use. Blood Cells Mol Dis.

[19] Castegnaro S (2011). Effect of platelet lysate on the functional and molecular characteristics of mesenchymal stem cells isolated from adipose tissue. Curr Stem Cell Res Ther.

[20] Saeed AI (2003). Tm4: a free, open-source system for microarray data management and analysis. Biotechniques.

[21] Wegmeyer H (2013). Mesenchymal stromal cell characteristics vary depending on their origin. Stem Cells Dev.

[22] Libro R (2016). Cannabidiol modulates the immunophenotype and inhibits the activation of the inflammasome in human gingival mesenchymal stem cells. Front Physiol.

[23] Cantor JM, Ginsberg MH (2012). Cd98 at the crossroads of adaptive immunity and cancer. J Cell Sci.

[24] Josson S (2011). B2-microglobulin induces epithelial to mesenchymal transition and confers cancer lethality and bone metastasis in human cancer cells. Cancer Res.

[25] Xu J (2013). A novel role of Emmprin/Cd147 in transformation of quiescent fibroblasts to cancer-associated fibroblasts by breast cancer cells. Cancer Lett.

[26] Veenstra RG (2015). B7-H3 expression in donor t cells and host cells negatively regulates acute graft-versus-host disease lethality. Blood.

[27] Okolicsanyi RK (2015). Human mesenchymal stem cells retain multilineage differentiation capacity including neural marker expression after extended in vitro expansion. Plos One.

[28] Camilleri ET (2016). Identification and validation of multiple cell surface markers of clinical-grade adipose-derived mesenchymal stromal cells as novel release criteria for good manufacturing practice-compliant production. Stem Cell Res Ther.

[29] Ong WK (2014). Identification of specific cell-surface markers of adipose-derived stem cells from subcutaneous and visceral fat depots. Stem Cell Rep.

[30] Bernstein KE (2013). A modern understanding of the traditional and nontraditional biological functions of angiotensin-converting enzyme. Pharmacol Rev.

[31] Park TS, Zambidis ET (2009). A role for the renin-angiotensin system in hematopoiesis. Haematologica.

[32] Rodgers KE, Dizerega GS (2013). Contribution of the local RAS to hematopoietic function: a novel therapeutic target. Front Endocrinol (Lausanne).

[33] Haznedaroglu IC, Beyazit Y (2013). Local bone marrow renin-angiotensin system in primitive, definitive and neoplastic haematopoiesis. Clin Sci (Lond).

[34] Zambidis ET (2008). Expression of angiotensin-converting enzyme (CD143) identifies and regulates primitive hemangioblasts derived from human pluripotent stem cells. Blood.

[35] Ramshaw HS (2001). Monoclonal antibody BB9 raised against bone marrow stromal cells identifies a cell-surface glycoprotein expressed by primitive human hemopoietic progenitors. Exp Hematol.

[36] Jokubaitis VJ (2008). Angiotensin-converting enzyme (CD143) marks hematopoietic stem cells in human embryonic, fetal, and adult hematopoietic tissues. Blood.

[37] Sinka L (2012). Angiotensin-converting enzyme (CD143) specifies emerging lympho-hematopoietic progenitors in the human embryo. Blood.

[38] Matsushita K (2006). Local renin angiotensin expression regulates human mesenchymal stem cell differentiation to adipocytes. Hypertension.

[39] Matsushita K (2016). Deletion of angiotensin II type 2 receptor accelerates adipogenesis in murine mesenchymal stem cells via Wnt10b/beta-catenin signaling. Lab Invest.

[40] Silva EA, Eseonu C, Mooney DJ (2014). Endothelial cells expressing low levels of CD143 (ACE) exhibit enhanced sprouting and potency in relieving tissue ischemia. Angiogenesis.

