# Supplementary figures and images for: High-throughput immunophenotypic characterization of bone marrow- and cord blood-derived mesenchymal stromal cells reveals common and differentially expressed markers: identification of angiotensin-converting enzyme (CD143) as a marker differentially expressed between adult and perinatal tissue sources
Source: Stem Cell Res Ther. 2018 Jan 16;9:10. doi: 10.1186/s13287-017-0755-3 (PMC5771027; doi:10.1186/s13287-017-0755-3)

a)

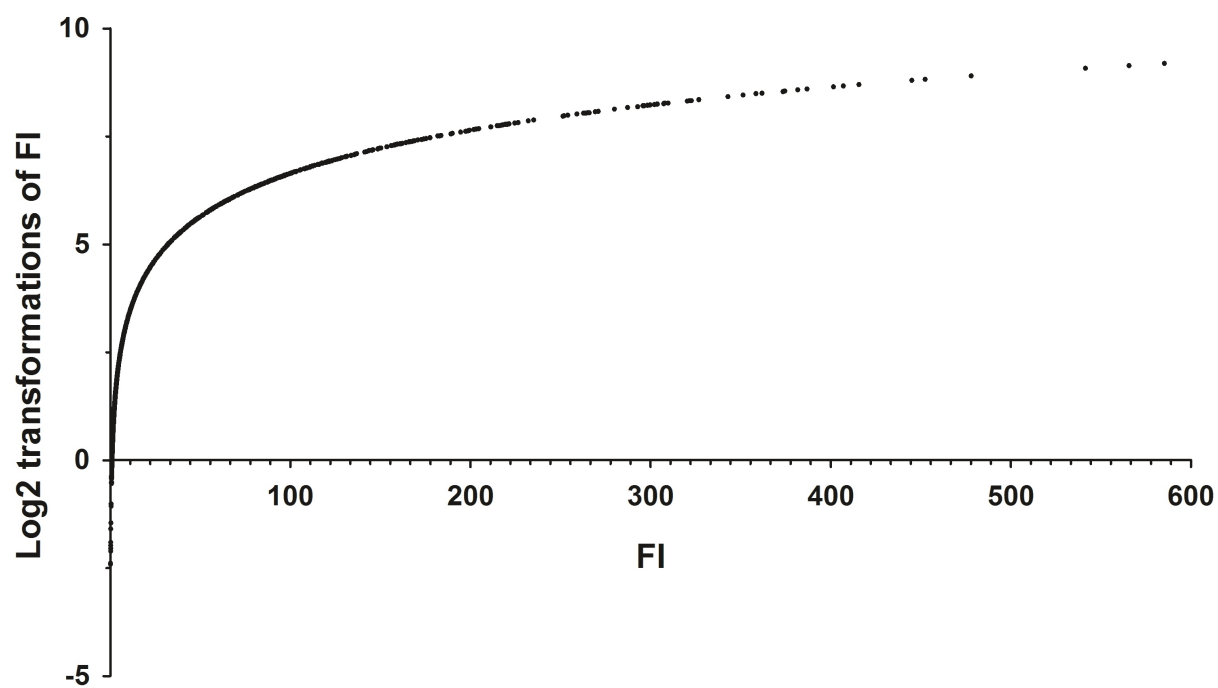

b)

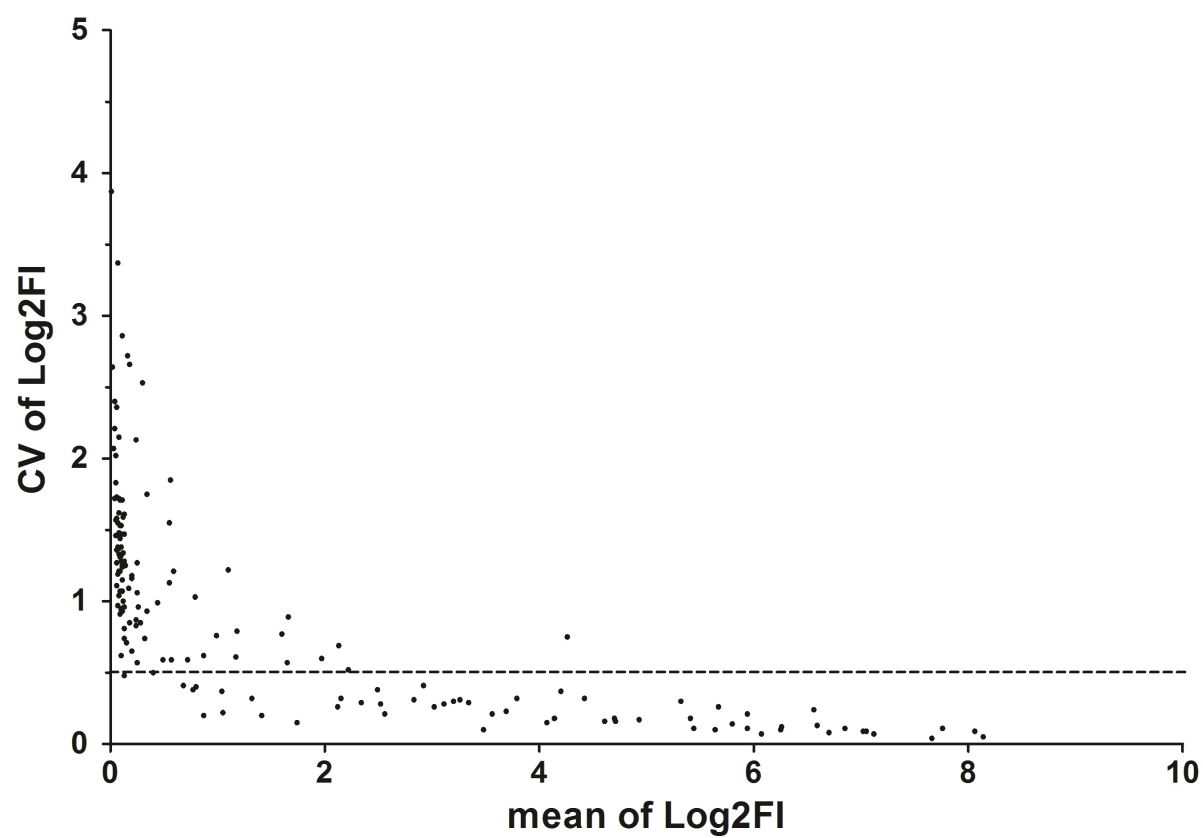

Supplement: Supplementary file 1 — Transformation and selection. a) The log2 transformation of FI was performed to improve the resolution of low FI values. b) The markers with the smallest variability across samples independently from the source were selected according to a CV of log2 FI ≤ 0.5. Accordingly, 59 markers represented below the dotted line were subsequently considered for unsupervised hierarchical cluster analysis. (PDF 439 kb) [file 13287_2017_755_MOESM1_ESM.pdf]

## Slide 1
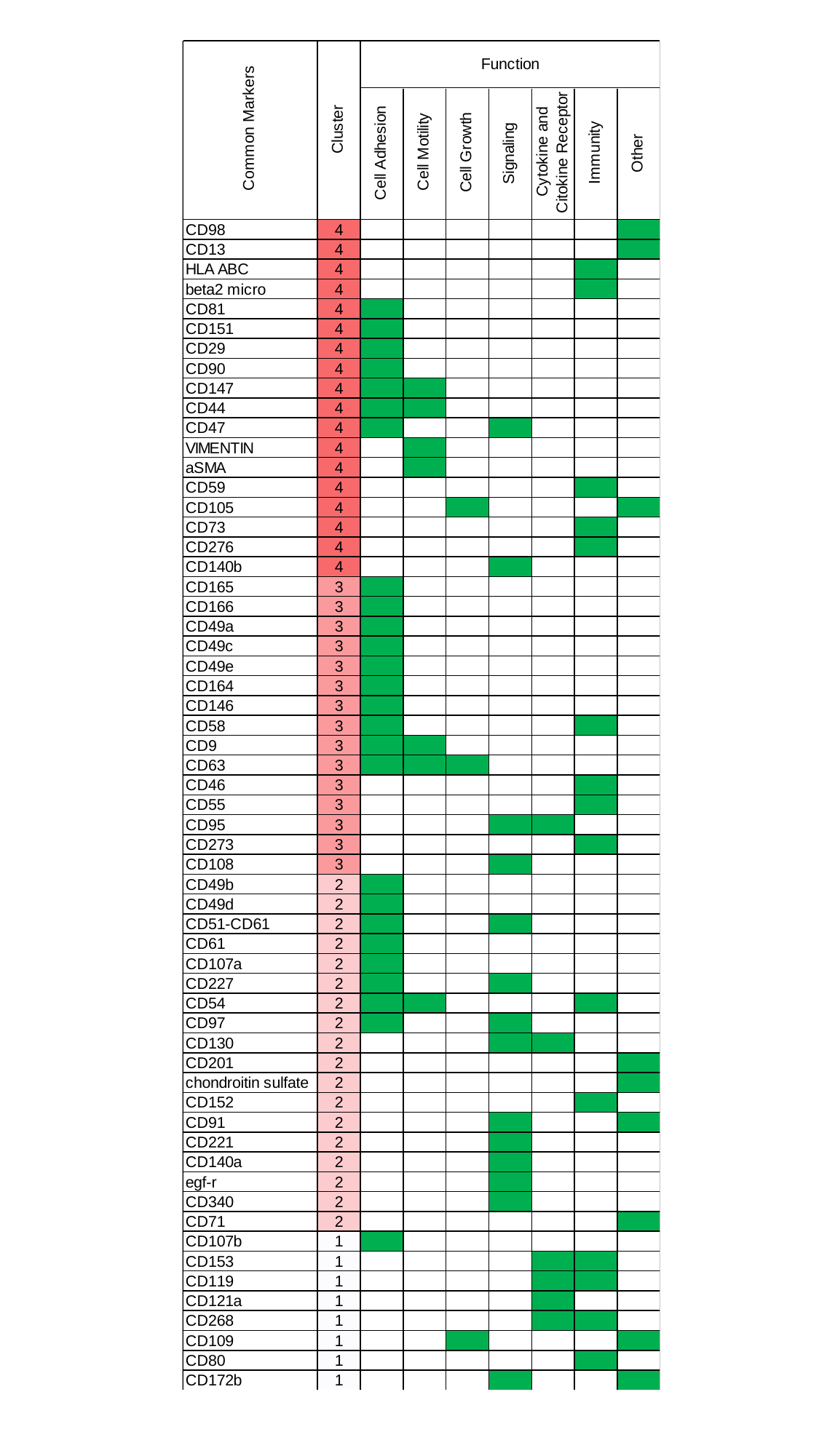

Supplement: Supplementary file 3 — List of the lowest variable markers (n = 59, CV cut-off of log2 FI ≤ 0.5) sorted by function and cluster expression. Markers with no expression were assigned to cluster 1, low-expressed markers to cluster 2, and intermediate markers to cluster 3, while a total of 18 markers clustering as very strong were assigned to cluster 4. (PPTX 50 kb) [file 13287_2017_755_MOESM3_ESM.pptx]

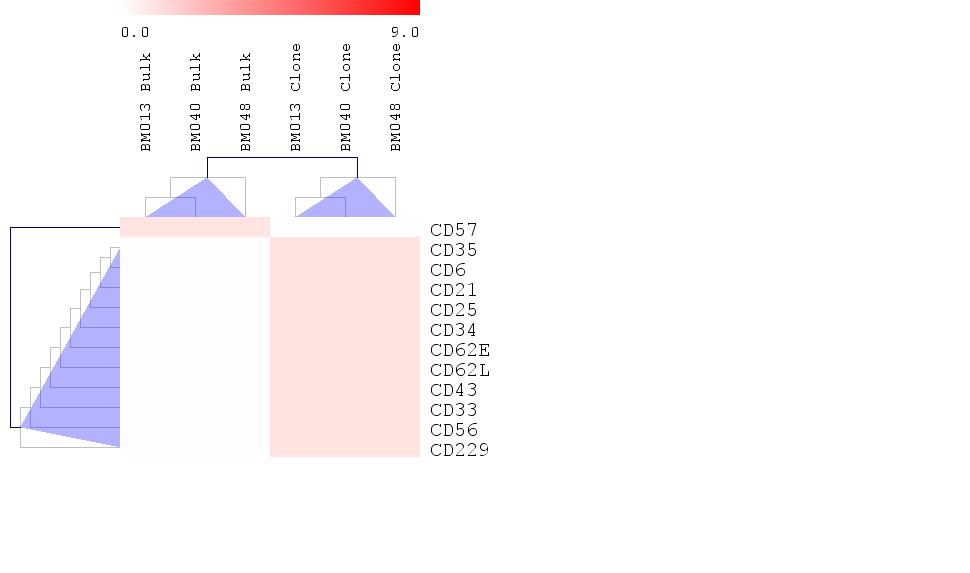

Supplement: Supplementary file 4 — Differentially expressed markers between bulk cultures and single derived clones. The expression of each marker (n = 246) was compared by paired t test (n = 3; p < 0.01). (TIFF 1629 kb) [file 13287_2017_755_MOESM4_ESM.tiff]

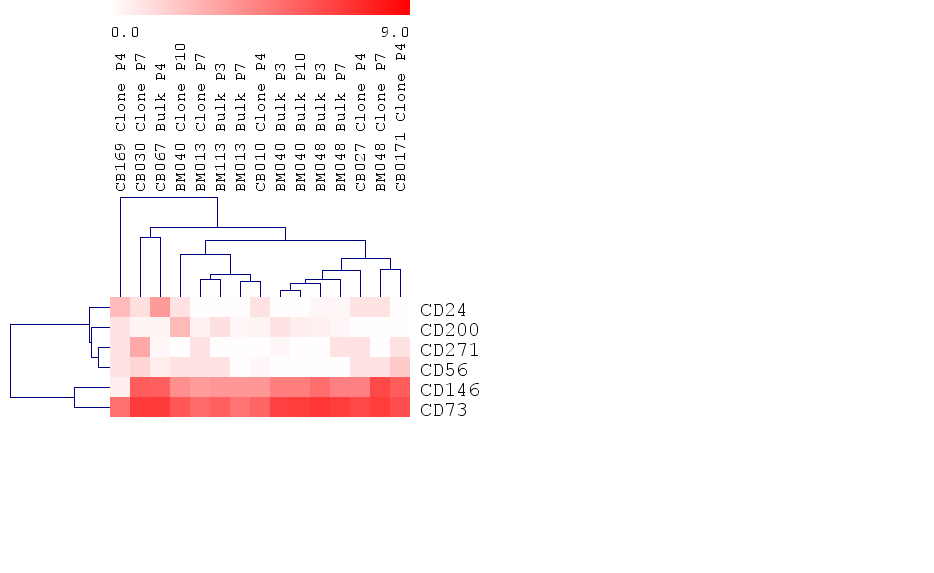

Supplement: Supplementary file 5 — Neural marker classification according to unsupervised HCL. Heat-map expression of the markers identified by Okolicsanyi [27]: CD24, CD200, CD271, CD56, CD146, and CD73. Antigen expression is color coded from white (no expression) to red (strong expression). Data are presented as log2 FI (median fluorescence intensity on the isotype control). (TIFF 1568 kb) [file 13287_2017_755_MOESM5_ESM.tiff]
