# Supplementary material for: High-throughput immunophenotypic characterization of bone marrow- and cord blood-derived mesenchymal stromal cells reveals common and differentially expressed markers: identification of angiotensin-converting enzyme (CD143) as a marker differentially expressed between adult and perinatal tissue sources
Source: Stem Cell Res Ther. 2018 Jan 16;9:10. doi: 10.1186/s13287-017-0755-3 (PMC5771027; doi:10.1186/s13287-017-0755-3)
Supplement: Supplementary file 2 — Mean and CV values of log2 FI for each of 246 investigated markers. (DOCX 22 kb) [file 13287_2017_755_MOESM2_ESM.docx]

**Table S1: Mean and CV values of Log2FI for each of 246 markers**

| **Low variation markers (CV of Log2FI≤0.5)** | | | **High variation markers (CV of Log2FI>0.5)** | | |
| --- | --- | --- | --- | --- | --- |
| **Marker** | **Mean of Log2FI** | **CV of Log2FI** | **Marker** | **Mean of Log2FI** | **CV of Log2FI** |
| **CD81** | 7,66 | 0,04 | **CD49f** | 2,22 | 0,52 |
| **CD276** | 8,14 | 0,05 | **CD274** | 1,65 | 0,57 |
| **CD47** | 6,07 | 0,07 | **CD74** | 0,25 | 0,57 |
| **CD90** | 7,12 | 0,07 | **CD11a** | 0,49 | 0,59 |
| **CD98** | 6,7 | 0,08 | **CD162** | 0,72 | 0,59 |
| **HLA class I** | 7,05 | 0,09 | **HLA DQ** | 0,57 | 0,59 |
| **CD147** | 7,02 | 0,09 | **CD99** | 1,97 | 0,6 |
| **CD44** | 8,06 | 0,09 | **CD10** | 1,17 | 0,61 |
| **beta2micro** | 5,64 | 0,1 | **CD26** | 0,87 | 0,62 |
| **CD151** | 6,25 | 0,1 | **CD178** | 0,1 | 0,62 |
| **CD340** | 3,48 | 0,1 | **CD84** | 0,2 | 0,65 |
| **Vimentin** | 7,76 | 0,11 | **SSEA_4** | 2,13 | 0,69 |
| **CD46** | 5,44 | 0,11 | **CD19** | 0,15 | 0,71 |
| **CD29** | 5,94 | 0,11 | **CD14** | 0,32 | 0,74 |
| **CD105** | 6,85 | 0,11 | **CD154** | 0,13 | 0,74 |
| **CD59** | 7,76 | 0,11 | **HLA A2** | 4,26 | 0,75 |
| **CD73** | 6,26 | 0,12 | **CD141** | 0,99 | 0,76 |
| **CD13** | 6,59 | 0,13 | **Hem Pr Cell** | 1,6 | 0,77 |
| **CD166** | 5,8 | 0,14 | **CD106** | 1,18 | 0,79 |
| **CD58** | 4,07 | 0,15 | **CD124** | 0,13 | 0,81 |
| **CD130** | 1,74 | 0,15 | **CD31** | 0,13 | 0,81 |
| **CD55** | 4,71 | 0,16 | **fMLR_P** | 0,24 | 0,83 |
| **CD164** | 4,61 | 0,16 | **CD120a** | 0,28 | 0,85 |
| **CD95** | 4,93 | 0,17 | **CD163** | 0,18 | 0,85 |
| **CD165** | 4,14 | 0,18 | **CD18** | 0,24 | 0,87 |
| **CD49e** | 5,41 | 0,18 | **Digang GD2** | 1,66 | 0,89 |
| **CD63** | 4,7 | 0,18 | **CD180** | 0,09 | 0,91 |
| **CD107b** | 0,87 | 0,2 | **CD41a** | 0,09 | 0,91 |
| **CD107a** | 1,41 | 0,2 | **CD209** | 0,11 | 0,93 |
| **EGF_R** | 3,56 | 0,21 | **CD112** | 0,34 | 0,93 |
| **CD49d** | 2,56 | 0,21 | **CD83** | 0,1 | 0,94 |
| **CD140b** | 5,94 | 0,21 | **CD7** | 0,1 | 0,94 |
| **CD119** | 1,05 | 0,22 | **CD57** | 0,11 | 0,95 |
| **CD273** | 3,69 | 0,23 | **CD321** | 0,11 | 0,95 |
| **aSMA** | 6,56 | 0,24 | **CDw93** | 0,13 | 0,96 |
| **CD49c** | 5,67 | 0,26 | **CD4** | 0,26 | 0,96 |
| **CD221** | 2,12 | 0,26 | **CD94** | 0,07 | 0,97 |
| **CD49b** | 3,02 | 0,26 | **CD4v4** | 0,44 | 0,99 |
| **CD140a** | 3,11 | 0,28 | **CD326** | 0,12 | 1 |
| **Chondroitin** | 2,52 | 0,28 | **CD30** | 0,12 | 1 |
| **CD227** | 3,34 | 0,29 | **CD102** | 0,79 | 1,03 |
| **CD91** | 2,34 | 0,29 | **CD127** | 0,08 | 1,04 |
| **CD51_61** | 3,2 | 0,3 | **CD35** | 0,08 | 1,04 |
| **CD108** | 5,32 | 0,3 | **CD42a** | 0,08 | 1,04 |
| **CD201** | 3,26 | 0,31 | **CD177** | 0,08 | 1,04 |
| **CD61** | 2,83 | 0,31 | **CD11c** | 0,08 | 1,04 |
| **CD152** | 1,32 | 0,32 | **CD8b** | 0,08 | 1,04 |
| **CD71** | 2,15 | 0,32 | **CD123** | 0,25 | 1,06 |
| **CD9** | 3,79 | 0,32 | **CD1b** | 0,11 | 1,07 |
| **CD146** | 4,42 | 0,32 | **CD309** | 0,11 | 1,07 |
| **CD80** | 1,04 | 0,37 | **CD220** | 0,09 | 1,07 |
| **CD49a** | 4,2 | 0,37 | **CD314** | 0,09 | 1,07 |
| **CD54** | 2,49 | 0,38 | **HLA class II** | 0,17 | 1,09 |
| **CD109** | 0,77 | 0,38 | **CD6** | 0,06 | 1,11 |
| **CD172b** | 0,8 | 0,4 | **CD79b** | 0,06 | 1,11 |
| **CD97** | 2,92 | 0,41 | **CD200** | 0,55 | 1,13 |
| **CD121a** | 0,68 | 0,41 | **CD161** | 0,11 | 1,15 |
| **CD153** | 0,13 | 0,48 | **NKB1** | 0,11 | 1,15 |
| **CD268** | 0,4 | 0,5 | **CD88** | 0,2 | 1,16 |
|  |  |  | **CD40** | 0,2 | 1,18 |
|  |  |  | **CD103** | 0,07 | 1,19 |
|  |  |  | **CD138** | 0,07 | 1,19 |
|  |  |  | **CD21** | 0,07 | 1,19 |
|  |  |  | **CD305** | 0,07 | 1,19 |
|  |  |  | **CD337** | 0,07 | 1,19 |
|  |  |  | **CD39** | 0,59 | 1,21 |
|  |  |  | **CD8a** | 0,09 | 1,21 |
|  |  |  | **CD134** | 0,09 | 1,21 |
|  |  |  | **CD22** | 0,08 | 1,21 |
|  |  |  | **CMRF56** | 0,08 | 1,21 |
|  |  |  | **CD142** | 1,1 | 1,22 |
|  |  |  | **CD38** | 0,11 | 1,24 |
|  |  |  | **CD210** | 0,14 | 1,25 |
|  |  |  | **CD336** | 0,11 | 1,25 |
|  |  |  | **gdTCR** | 0,12 | 1,26 |
|  |  |  | **HLA DR** | 0,25 | 1,27 |
|  |  |  | **CD23** | 0,06 | 1,27 |
|  |  |  | **CD25** | 0,06 | 1,27 |
|  |  |  | **CD34** | 0,06 | 1,27 |
|  |  |  | **CD42b** | 0,06 | 1,27 |
|  |  |  | **CD62E** | 0,06 | 1,27 |
|  |  |  | **CD62L** | 0,06 | 1,27 |
|  |  |  | **CD226** | 0,06 | 1,27 |
|  |  |  | **CD50** | 0,06 | 1,27 |
|  |  |  | **CD99R** | 0,11 | 1,27 |
|  |  |  | **abTCR** | 0,13 | 1,28 |
|  |  |  | **CDw329** | 0,1 | 1,28 |
|  |  |  | **CD205** | 0,13 | 1,28 |
|  |  |  | **CD118** | 0,09 | 1,31 |
|  |  |  | **CD16** | 0,09 | 1,31 |
|  |  |  | **CD1a** | 0,09 | 1,31 |
|  |  |  | **CD206** | 0,1 | 1,32 |
|  |  |  | **CD86** | 0,08 | 1,33 |
|  |  |  | **Integrin B7** | 0,12 | 1,34 |
|  |  |  | **CD126** | 0,06 | 1,36 |
|  |  |  | **CD100** | 0,06 | 1,36 |
|  |  |  | **CD137 ligand** | 0,06 | 1,36 |
|  |  |  | **CD45** | 0,06 | 1,36 |
|  |  |  | **CD244** | 0,06 | 1,36 |
|  |  |  | **CD85** | 0,06 | 1,36 |
|  |  |  | **CD32** | 0,08 | 1,37 |
|  |  |  | **CD120b** | 0,1 | 1,38 |
|  |  |  | **CD196** | 0,07 | 1,38 |
|  |  |  | **CD28** | 0,07 | 1,38 |
|  |  |  | **CD338** | 0,07 | 1,38 |
|  |  |  | **CD2** | 0,07 | 1,38 |
|  |  |  | **Inv NKT** | 0,07 | 1,38 |
|  |  |  | **CDw328** | 0,09 | 1,44 |
|  |  |  | **CD243** | 0,05 | 1,46 |
|  |  |  | **CD53** | 0,05 | 1,46 |
|  |  |  | **CD181** | 0,05 | 1,46 |
|  |  |  | **CD121b** | 0,05 | 1,46 |
|  |  |  | **CD43** | 0,05 | 1,46 |
|  |  |  | **CD267** | 0,13 | 1,47 |
|  |  |  | **CD335** | 0,1 | 1,47 |
|  |  |  | **CD77** | 0,11 | 1,47 |
|  |  |  | **CLIP** | 0,08 | 1,48 |
|  |  |  | **CD66f** | 0,08 | 1,48 |
|  |  |  | **CD69** | 0,08 | 1,48 |
|  |  |  | **CD15** | 0,08 | 1,48 |
|  |  |  | **CD5** | 0,08 | 1,48 |
|  |  |  | **CD3** | 0,09 | 1,53 |
|  |  |  | **CDw327** | 0,1 | 1,53 |
|  |  |  | **M_CA_B** | 0,55 | 1,55 |
|  |  |  | **CMRF44** | 0,07 | 1,55 |
|  |  |  | **CD135** | 0,05 | 1,57 |
|  |  |  | **CD137** | 0,05 | 1,57 |
|  |  |  | **CD27** | 0,05 | 1,57 |
|  |  |  | **CD33** | 0,05 | 1,57 |
|  |  |  | **CLA** | 0,05 | 1,57 |
|  |  |  | **Vbeta23** | 0,05 | 1,57 |
|  |  |  | **CD158b** | 0,05 | 1,57 |
|  |  |  | **CD278** | 0,05 | 1,57 |
|  |  |  | **CD41b** | 0,05 | 1,57 |
|  |  |  | **SSEA_1** | 0,06 | 1,58 |
|  |  |  | **CD45RB** | 0,12 | 1,59 |
|  |  |  | **CD294** | 0,13 | 1,61 |
|  |  |  | **BLTR_1** | 0,08 | 1,62 |
|  |  |  | **SSEA_3** | 0,11 | 1,71 |
|  |  |  | **CD36** | 0,09 | 1,71 |
|  |  |  | **CD117** | 0,04 | 1,72 |
|  |  |  | **CD184** | 0,04 | 1,72 |
|  |  |  | **CD64** | 0,04 | 1,72 |
|  |  |  | **CD72** | 0,04 | 1,72 |
|  |  |  | **CD144** | 0,04 | 1,72 |
|  |  |  | **CD231** | 0,04 | 1,72 |
|  |  |  | **CD279** | 0,04 | 1,72 |
|  |  |  | **CD89** | 0,08 | 1,72 |
|  |  |  | **CD150** | 0,06 | 1,73 |
|  |  |  | **CD87** | 0,06 | 1,73 |
|  |  |  | **CD56** | 0,34 | 1,75 |
|  |  |  | **CD183** | 0,05 | 1,83 |
|  |  |  | **CD195** | 0,05 | 1,83 |
|  |  |  | **CD104** | 0,05 | 1,83 |
|  |  |  | **CD11b** | 0,05 | 1,83 |
|  |  |  | **CD193** | 0,05 | 1,83 |
|  |  |  | **CD1d** | 0,05 | 1,83 |
|  |  |  | **CD282** | 0,05 | 1,83 |
|  |  |  | **CD275** | 0,05 | 1,83 |
|  |  |  | **CD37** | 0,05 | 1,83 |
|  |  |  | **CD24** | 0,56 | 1,85 |
|  |  |  | **CD132** | 0,05 | 2,02 |
|  |  |  | **CD45RA** | 0,03 | 2,07 |
|  |  |  | **CD66 acde** | 0,03 | 2,07 |
|  |  |  | **TRA_1_60** | 0,03 | 2,07 |
|  |  |  | **CD229** | 0,03 | 2,07 |
|  |  |  | **CD62P** | 0,03 | 2,07 |
|  |  |  | **CD171** | 0,03 | 2,07 |
|  |  |  | **CD48** | 0,03 | 2,07 |
|  |  |  | **CD114** | 0,03 | 2,07 |
|  |  |  | **CD45RO** | 0,24 | 2,13 |
|  |  |  | **CD212** | 0,08 | 2,15 |
|  |  |  | **CD255** | 0,04 | 2,21 |
|  |  |  | **CD235a** | 0,04 | 2,21 |
|  |  |  | **CD116** | 0,06 | 2,36 |
|  |  |  | **CD15s** | 0,04 | 2,4 |
|  |  |  | **CD271** | 0,3 | 2,53 |
|  |  |  | **CD158a** | 0,02 | 2,64 |
|  |  |  | **CD197** | 0,02 | 2,64 |
|  |  |  | **CD20** | 0,02 | 2,64 |
|  |  |  | **CD66b** | 0,02 | 2,64 |
|  |  |  | **CD70** | 0,18 | 2,66 |
|  |  |  | **CD122** | 0,16 | 2,72 |
|  |  |  | **CD128b** | 0,11 | 2,86 |
|  |  |  | **CD75** | 0,07 | 3,37 |
|  |  |  | **TRA_1_81** | 0,01 | 3,87 |
|  |  |  | **Vbeta8** | 0,01 | 3,87 |
